# Modified DCF (Docetaxel, Cisplatin and 5-fluorouracil) chemotherapy is effective for the treatment of advanced rectal squamous cell carcinoma

**DOI:** 10.3389/fonc.2022.974108

**Published:** 2022-11-17

**Authors:** Laure Hervé, Stefano Kim, Jihane Boustani, Elodie Klajer, Mandy Pernot, Thierry Nguyen, Zaher Lakkis, Christophe Borg, Angélique Vienot

**Affiliations:** ^1^ Department of Medical Oncology, University Hospital of Besançon, Besançon, France; ^2^ INSERM, EFS BFC, RIGHT, University of Bourgogne Franche-Comté, Interactions Greffon-Hôte-Tumeur/Ingénierie Cellulaire et Génique, Besançon, France; ^3^ Clinical Investigational Center, CIC-1431, Besançon, France; ^4^ Department of Medical Oncology, Sanatorio Allende, Cordoba, Argentina; ^5^ Department of Radiotherapy, University Hospital of Besançon, Besançon, France; ^6^ Department of Digestive Surgery and Liver Transplantation, University Hospital of Besançon, Besançon, France

**Keywords:** squamous cell carcinoma, rectal cancer, polychemotherapy, DCF, chemoradiotherapy

## Abstract

**Background:**

Advanced rectal squamous cell carcinoma (rSCC) is a very rare and aggressive entity, and the best initial management is crucial for long survival as well as organ preservation and quality of life. Whereas local diseases are treated with chemo-radiotherapy and salvage surgery, data are scarce on how to treat more advanced diseases, and the role of induction chemotherapy is unknown.

**Methods:**

We retrospectively analyzed all consecutive patients with advanced rSCC and treated with modified DCF (docetaxel, cisplatin, 5-fluorouracil; mDCF) regimen, from January 2014 and December 2021 in two French centers. Exploratory endpoints were efficacy (overall survival, recurrence-free survival, response rate, organ preservation rate) and safety.

**Results:**

Nine patients with locally advanced or metastatic diseases received a mDCF regimen and were included for analysis. The median age was 62.0 years, 7 patients (77.8%) were women, and all eight available tumors were positive for HPV, mostly (85.7%) to genotype 16. With a median follow-up of 33.1 months, 77.8% of patients were still alive and disease-free, and the median overall survival was not reached at six years. The objective response rate was 87.5% after mDCF, and the complete response rate was 25.0% after mDCF and was increased to 75.0% after chemoradiotherapy. Only one patient underwent surgery on the primary tumor, with a complete pathological response. The median mDCF cycle was eight over eight scheduled, and all patients received the complete dose of radiotherapy without interruptions.

**Conclusions:**

Induction mDCF chemotherapy followed by chemoradiotherapy is safe and highly effective in patients with advanced rSCC, and should be considered as an option in metastatic stage or locally advanced disease with an organ-preservation strategy.

## Introduction

Squamous cell carcinoma (SCC) located in the rectum is a rare entity encompassing nearly 0.3% of rectal cancers. Rectal SCC (rSCC) was first described in the literature in 1933 by Raiford et al. (1), closely following the SCC subtype in the colon epithelium described in 1919 by Schmidtmann et al. (2). rSCC occurs mostly in females with a median age of 60 years.

Several possible hypotheses were drawn to clarify the presence of SCC in the rectal epithelium. The development of epithelial metaplasia might occur in response to several chronic inflammations such as inflammatory bowel disease (3–5), schistosomiasis, and amoebiasis (6) or a history of pelvic radiotherapy (7, 8). This hypothesis was explored *in vivo* by Reeve et al. in 1975 (9). Using chronic exposition to inflammatory chemical agents, the authors reported the appearance of epithelial metaplasia in rats’ colons.

The possible existence of pluripotent stem cells capable of a multidirectional differentiation in the colorectal mucosa has been suggested since the 1950s by several authors (10–12). This hypothesis was later supported by Nahas et al. (13) after studying the cytokeratin profile of five samples of rSCC and ten samples of anal SCC (aSCC) as controls. Distinction from aSCC can be difficult, but can be facilitated by immunohistochemical staining for cytokeratins (14, 15). Another hypothesis is the transformation of adenosquamous polyps into adenosquamous carcinomas (16, 17).

Several cases of rSCC occurring in patients with human immunodeficiency virus (HIV) have also been reported in the literature (18, 19). This association was further detailed by Coghill et al. (20) in a retrospective study including 1189 cases of colorectal cancers (adenocarcinomas and SCC) between 1991 and 2010 in HIV-infected patients. rSCC accounted for approximately 30% of rectal cancers in HIV-infected patients, representing an increase of the relative risk (RR) by about a factor of 30 (RR=28.9 [23.2-35.6]) compared to the overall population. An increased risk of rSCC compared to the overall population was also reported in this survey in solid organ transplant patients (RR=3.86 [1.66-6.36]).

There is therefore an increased risk of rSCC in these two immunocompromised populations, which raises the question of a causal role for human papillomavirus (HPV) infection. However, there are only a limited number of studies that have explored the involvement of HPV infections in rSCC (10, 19, 21–23). Coghill et al. (23) analyzed 24 pathological specimens of rSCC, 11 specimens of rectal adenocarcinomas (rADK), and 11 specimens of aSCC for the presence of HPV-16 using polymerase chain reaction (PCR), reverse transcriptase-PCR (RT-PCR) and *in situ* hybridization (ISH) for each specimen (**Table 1**).

**Table 1:** Presence of HPV-16 by histology and detection technique in the Coghill study (23).

	rSCC	rADK	aSCC
**PCR**	63%	0%	63%
**RT PCR**	78%	0%	56%
**ISH**	71%	0%	60%

rSCC, rectal squamous cell carcinoma; rADK, rectal adenocarcinoma; aSCC, anal squamous cell carcinoma; PCR, Polymerase Chain Reaction; RT-PCR, Reverse Transcriptase PCR; ISH, In Situ Hybridization.

On the other hand, there are several negative studies, such as that of Audeau et al. in 2002 (24) which showed no positive results after testing for HPV 6, 11, 16, and 18 subtypes in immunohistochemistry on 20 anatomical specimens. Nahas et al. in 2007 and Frizelle et al. in 2001 also found negative results on five and six anatomical specimens respectively analyzed by ISH (13–25).

The symptoms at diagnosis are early and identical to those of rADK. This explains the discovery of localized (52.8%) or locally advanced (29.3%) stages in more than 80.0% of the cases (26, 27). Despite diagnosis at an early stage, the morbidity and mortality rates remain high, with a five-year survival rate of 48.9%. Indeed, these results are worse than the survival known to be related to localized or locally advanced aSCC or rADK (69.0% and 62.1% respectively), regardless of the stage (27, 28). This phenomenon is even more pronounced in advanced diseases, as shown in **Table 2**.

**Table 2:** Comparison of five-year survival by stage for rectal squamous cell carcinoma, anal squamous cell carcinoma and rectal adenocarcinoma based on data from the Astaras study (27).

OS at five years	rADK	aSCC	rSCC
**All stages**	62.1%	69%	48.9%
**Stages I-II**	91.8%	82%	73.1%
**Stage III**	65.8%	65%	31.3%
**Stage IV**	20.8%	32%	8.8%

OS, overall survival; rADK, rectal adenocarcinoma; aSCC, anal squamous cell carcinoma; rSCC, rectal squamous cell carcinoma.

### Treatment of rSCC: A literature review

While the risk of local recurrences and distant metastases promoted the development of total neoadjuvant therapy in rADK (29), the treatment for locally advanced rSCC remains chemoradiotherapy (CRT) followed by salvage surgery in case of an incomplete response (30). Surgery alone (26) or combined CRT and surgery (31, 32) failed to demonstrate superiority over CRT. In a review of the literature from 1946 to 2015, Guerra et al. (26) found an improvement in overall survival (OS) in the CRT group compared to surgery alone: 86% versus 48%. There was 60% of complete radiological response rate after CRT and more than one complete pathological response out of two in the 44% of patients who underwent surgery. There was also a reduction in the rate of metastatic relapses, 13% after CRT versus 30% after surgery. Similarly, Kommalapati et al. (33), in a retrospective study including more than 3000 cases of rSCC (all stages) from the SEER database between 2004 and 2015, showed an increase in OS for stages II and III: median OS increased from 76 months for patients treated with surgery alone to 108 months for those treated with CRT (p=0.012). These data were not significant for stage I patients.

In order to better define the tumor response rates expected following CRT, we reviewed twelve case series reported in the literature, including a total of 103 patients treated by CRT for a localized or locally advanced rSCC (**Table 3**; 13, 34–44). An objective response rate (ORR) of 93% was reported after CRT for all stages and 74 out of the 103 assessable patients achieved a complete response (72%). The ORR was 95% for stage III (39 patients out of 41 assessable patients) and the complete response rate (CRR) was 57% (24 out of 42 assessable patients).

**Table 3:** Review of literature, patients with rectal squamous cell carcinoma treated with chemoradiotherapy and salvage surgery.

Authors	N	CRT (Gy)	Concurrent chemotherapy	ORR	CRR	CRR stage III	Salvage surgery rates	pCR	Relapse	OS
*Nahas et al.* 2007 (13)	9	50.4	- 3 5FU + CDDP (33%) - 6 5FU + MMC (67%)	NA	2 (22%)	0	7 (78%)	4 (80%)	0	100% at 30 months
*Clark et al.* 2008 (34)	7	50.4	- 3 5FU + MMC (43%) - 4 5FU or capecitabine + CDDP (57%)	7 (100%)	6 (86%)	5 (83%)	1 (14%)	1 (100%)	0	100% at 18 months
*Rasheed et al.* 2009 (35)	6	45- 50.4	- 2 5FU + MMC (33%) - 4 5FU + CDDP (67%)	6 (100%)	5 (83%)	4 (80%)	1 (17%)	1 (100%)	1 LR (16.6%)	100% at 5 years
*Tronconi et al.* 2010 (36)	6	50.4 - 59.4	- 4 5FU + CDDP (67%) - 1 5FU + MMC (16.6%) - 1 5FU seul (16,6%)	6 (100%)	4 (67%)	1 (33%)	3 (50%)	1 (17%)	1 LR et M+ (17%)	83% at 39 months
*Wang et al.* 2011 (37)	5	45- 54	- 5 5FU + MMC (100%)	5 (100%)	4 (80%)	1 (100%)	3 (60%)	3 (100%)	2 M+ (40%)	NA
*Yeh et al.* 2012 (38)	5	30- 60	- 4 5FU + MMC (80%) - 1 5FU + CDDP (20%)	5 (100%)	4 (80%)	2 (100%)	1 (20%)	1 (100%)	1 M+ (20%)	80% at 44 months
*Jeong et al.* 2013 (39)	4	50.4 - 63	- 4 5FU or capecitabine + CDDP (100%)	3* (75%)	3 (75%)	2 (67%)	0 (0%)	NA	0	75% at 5 years
*Peron et al.* 2015 (40)	11	45 - 62	- 5 5FU + CDDP (50%) - 4 5FU + MMC (40%) - 1 capecitabine (10%)	11 (100%)	7 (63%)	5 (55%)	4 (36%)	2 (50%)	1 LR + 1 M+ (18%)	100% at 56 months
*Musio et al.* 2015 (41)	8	45 - 70	- 6 5FU + MMC (75%) - 2 ralitrexed + oxaliplatine (25%)	7 (87.5%)	6 (75%)	4 (67%)	2 (25%)	NA	1 LR (12.5%)	88% at 42 months
*Loganadane et al.* 2016 (42)	23	45 -65	- 12 5FU + CDDP (54%) - 8 5FU ou capecitabine + MMC (36%) - 2 CDDP (10%)	21 (91%)	19 (83%)	NA	4 (17%)	2 (50%)	2 LR + 2 M+ 1 LR and M+ (22%)	86% at 5 years
*Sturgeon et al.* 2017 (43)	14	38 - 58	- 14 5FU ou capecitabine + CDDP (100%)	12 (86%)	12 (86%)	NA	2 (14%)	0	2 LR (14%)	86% at 5 years
*Song et al.* 2020 (44)	5	50 - 54	NA	NA	2 (40%)	NA	0% (0%)	NA	1 LR + 1 M+ 1 LR and M+ (60%)	NA

N, number of patients; CRT, chemoradiotherapy; ORR, Objective Response Rate; CRR, Complete Response Rate; pCR, pathological complete response; OS, Overall Survival; 5FU, 5-fluorouracil; CDDP, cisplatin; MMC, Mitomycin C; LR, local recurrence; M+, metastatic recurrence; NA, Not Applicable.

*4th patient: toxic death at two months from septic shock and febrile neutropenia.

The prognostic value of complete response is another major issue to better discriminate patients’ risk of relapse and death. In a study including more than 900 cases of localized aSCC treated with CRT ± maintenance chemotherapy, Glynne Jones et al. (45) showed a strong association between CRR following CRT and five-year OS. Five-year OS was 87% in patients with a complete response versus 46% in patients with an incomplete response (HR=0.17 [0.12-0.23]; p <0.0001). Similar exploratory analyses are warranted for rSCC.

### Rational for docetaxel, cisplatin and 5 fluorouracil (DCF) polychemotherapy

The management of locally advanced, unresectable, or metastatic rSCC remains a challenge. The role of upfront chemotherapy in advanced rSCC was never investigated. DCF is a multi-drug therapy consisting of docetaxel, cisplatin, and 5-fluorouracil. We have previously reported the high level of efficacy of DCF therapy in advanced aSCC in the Epitopes-HPV02 study (46). In this study, almost 50% of aSCC patients achieved a complete response and more than 80% of objective responses were reported.

Compared to standard DCF, modified DCF (mDCF) is administered every 14 days, intravenously, with a lower dose intensity for docetaxel and cisplatin (20mg/m^2^ per week versus 25mg/m^2^ per week) and a similar dose intensity for 5-fluorouracil (1200mg/m^2^ per week versus 1250mg/m^2^ per week). The main benefit was similar efficacy while mDCF allowed a better safety profile. In the Epitopes-HPV02 trial (46), 70% of grade 3 and 4 side effects were reported: 83% in the standard DCF group and 53% in the mDCF group. There were 14% febrile neutropenia in the standard DCF arm versus 0% in the mDCF arm. Altogether, treatment with mDCF generates a high level of long-lasting remissions in aSCC. However, the clinical interest of the DCF regimen was never investigated in rSCC. Here, we report the clinical results of a cohort of patients with advanced and unresectable rSCC treated with upfront mDCF.

## Materials and methods

### Patients

All consecutive patients with histologically proven rSCC who were treated with mDCF chemotherapy in two French hospitals (University Hospital of Besançon and North Franche-Comte Hospital) between January 2014 and December 2021 were included in this study. Patients with tumors involving the anal canal or the anorectal junction were excluded. All women underwent gynecologic examination to exclude a primary gynecologic tumor.

Demographics, cancer history, pathological, clinical, biological, and radiological (tumor response according to Response Evaluation Criteria in Solid Tumors [RECIST] v1.1 criteria) parameters at the beginning of mDCF treatment, as well as treatment outcomes, were retrospectively collected from medical records. The database was locked on 04/29/2021.

Immunohistochemistry staining (p53, p63, p40, CK20, CK7, CK8, CDX2, SATB2) was completed for the available samples. HPV genotyping in blood and tissue was also performed by PCR using the INNO-LiPA kit allowing the detection of 32 HPV subtypes belonging to the low-, potentially high-, and high-risk subgroups.

### Treatments

mDCF chemotherapy is a combination of docetaxel (40 mg/m^2^), followed by cisplatin (40 mg/m^2^) on Day 1. Then, a continuous intravenous infusion of 5-fluorouracil [5-FU] (2400 mg/m^2^) was administered over 46 h starting on Day-1 (46). This polychemotherapy was administered every two weeks, up to eight cycles, in a neoadjuvant setting.

Chemoradiotherapy (CRT) was then started between two and six weeks after the end of chemotherapy. Concomitant chemotherapy was given with capecitabine 1650mg/m^2^ daily, and mitomycin C in one to two injections (10 mg/m^2^). Target volumes were defined according to international guidelines. Tumor response was assessed by pelvic magnetic resonance imaging (MRI) after 5-7 cycles of chemotherapy, followed by a clinical examination including a digital rectal examination and pelvic MRI at least 6-8 weeks after the end of CRT. A biopsy was performed if an incomplete response or recurrence was suspected. In case of histologically proven recurrence, surgical resection was proposed at six to eight weeks after the end of preoperative treatment. Total mesorectal excision with sphincter preservation was carried out by using a conventional low rectal stapling anastomosis.

Patients were followed up every three months until five years after the end of treatments with computed tomography scan (CT-scan) and/or pelvic MRI, and clinical examination.

### Statistical analysis

Median value (interquartile range [IQR]) and frequency (percentage) were provided for the description of continuous and categorical variables, respectively. OS was calculated from the date of diagnosis to the date of death from any cause. Survival data were censored at the last follow-up. Recurrence-free survival (RFS) was calculated from the date of the end of specific treatments to the date of recurrence or death from any cause, or the date of the last follow-up, at which point data were censored. OS and RFS were estimated using the Kaplan-Meier method and described using median or rate at specific time points with 95% confidence intervals (CI), and compared using the log-rank test. ORR and CRR were determined according to RECIST v1.1 criteria. Toxicity was evaluated according to the National Cancer Institute Common Terminology Criteria (CTCAE v5). All analyses were performed using R software version 3.6.2 (R Development Core Team, Vienna, Austria; http://www.r-project.org). P<0.05 were considered statistically significant, and all tests were two-sided.

## Results

### Patient characteristics

From January 2014 to December 2021, nine rSCC patients were treated with mDCF chemotherapy and included in this cohort. Their characteristics are described in **Table 4**. The median age was 62.0 years (IQR, 57.0 – 66.0 years), seven patients (77.8%) were women, and all patients had a 0 or 1 performance status (ECOG-PS). No risk factor to develop rSCC was identified in these patients. In particular, all patients were HIV-negative. P16 overexpression was identified in all rSCC with available tumor samples (n=8). HPV genotyping on tumor tissue identified six (85.7%) HPV16 and one HPV18. The main symptoms at diagnosis were diarrhea/constipation with or without abdominal pain (77.8%), fecal urgency or tenesmus (44.4%), anorexia, and weight loss (33.3%). Patients treated with mDCF had locally advanced or rapidly progressing rSCC. Two patients showed synchronous metastases and one patient had metastatic relapse two years after CRT.

**Table 4:** Patient characteristics before mDCF chemotherapy.

Patient	Age (years)	Gender	ECOG-PS	p16 (HPV genotyping)	TNM	Primary Tumor size (mm)	Distance from anal sphincter (mm)	Metastatic sites
#1	74	Female	0	+ (HPV16)	T4N2M0	47	10	–
#2	57	Male	0	+ (HPV16)	T4N2M0	70	70	–
#3	58	Female	1	+ (HPV16)	T3N0M0	70	65	–
#4	63	Female	1	+ (HPV16)	T4N1M0	66	30	–
#5	72	Female	1	+ (HPV16)	T4N2M0	100	10	–
#6	50	Female	0	+ (NA)	T4NXM1	50	80	Synchronous peritoneal metastasis
#7	62	Male	1	+ (HPV18)	T4NXM1	150	10	Synchronous liver metastasis
#8	66	Female	0	NA (NA)	(T4N1M0 at diagnosis) Metastatic recurrence	63	80	Metachronous liver metastasis
#9	48	Female	0	+ (HPV16)	T4N2M0	70	70	–

ECOG-PS, Eastern Cooperative Oncology Group performance status; TNM, tumor, node, metastasis; NA, not available.

### mDCF chemotherapy

A median of eight cycles of the mDCF regimen was administered with a dose modification performed for 44.4% of the patients. In terms of safety, no grade ≥4 and no discontinuation of chemotherapy were reported. While 66.7% of patients had adverse events, grade 3 adverse events were reported in only two patients (asthenia or anorexia). Notably, hematopoietic growth factors were systematically used after each chemotherapy cycle from Day-3 to Day-7 for all patients. No febrile neutropenia occurred in our cohort (**Table 5**).

**Table 5:** mDCF administration, tumor responses and toxicities in patients with rectal squamous cell carcinoma.

Patient	mDCF administration	Cycles of mDCF delivered	Objective response rate	Dose modification	Adverse events (G: grade)
#1	Before CRT	6	Partial response	No	No
#2	Before CRT	8	Partial response	No	Asthenia (G1) Nausea G1
#3	Before CRT	5	Complete response	Yes 20% dose reduction for mDCF (Cycles 4-5)	Asthenia (G2) Nausea (G2)
#4	Before CRT	7	Partial response	Yes 20% dose reduction for mDCF (Cycle 7)	Asthenia (G1) Nausea G1
#5	Before CRT	8	Stable disease	No	Asthenia (G3) Nausea (G1)
#6	Before CRT	8	Complete response	No	No
#7	Before CRT	10	Partial response	No	Anorexia (G3)
#8	After CRT (relapse at two years)	8	Partial response	No	No
#9	Before CRT	6	Partial response	Yes 25% dose reduction for 5FU (Cycle 6)	Asthenia (G1) Hand-foot sydrome (G2)

CRT, chemoradiotherapy; mDCF, modified DCF chemotherapy; FOLFOX, oxaliplatin and 5-fluorouracil; 5FU, 5-fluorouracil.

mDCF treatment was used before CRT in a neoadjuvant setting for eight patients. The ORR achieved following mDCF was 87.5% and the CRR was 25.0%. No progression of rSCC disease occurred during treatment with neoadjuvant therapy.

Of note, patient #8 was exposed to the mDCF regimen for a recurrence after the first treatment by CRT. A chronic renal failure in this patient led to prescribe carboplatin instead of cisplatin. Interestingly, a partial response was observed by CT-scan after chemotherapy. Surgical resection with right hepatectomy was performed and a pathological complete response was confirmed.

### Chemoradiotherapy after mDCF neoadjuvant treatment

Eight patients received CRT after mDCF chemotherapy (excluding patient #8 previously exposed to CRT before mDCF initiation). The final ORR on the rectal carcinoma was 87.5% and the CRR was 75.0% (**Table 6**). Radiotherapy consisted in intensity modulated radiation therapy delivering total doses of 45 to 60 Gray in 1.8-2 Gray per fraction and no discontinuation was reported. Concomitant chemotherapy was discontinued for two patients: one for non-febrile neutropenia (#3) and one for cholestasis related to disease progression (#7). Dose modification of capecitabine was also reported for only one patient who experienced a grade 2 thrombocytopenia.

**Table 6:** Chemoradiotherapy modalities after mDCF neoadjuvant treatment in patients with rectal squamous cell carcinoma.

Patient	Gray	Concomitant chemotherapy	Objective response rate
#1	59.4	Capecitabine + MMC	Complete response
#2	59.4	Capecitabine + MMC	Complete response
#3	45	Capecitabine + MMC	Complete response
#4	59.4	Capecitabine + MMC	Complete response
#5	36	Capecitabine + MMC	Rectal progressive disease
#6	50	Capecitabine + MMC	Rectal complete response Peritoneal partial response
#7	60	Capecitabine + MMC	Rectal partial response Liver progressive disease
#9	50	Capecitabine	Complete response

MMC, mitomycin C.

### Tumor resection after mDCF and CRT combination

In the case of rectal partial response after CRT, surgical resection was proposed. One patient displayed also a liver metastasis progression after CRT, ruling out the indication of rectal tumor resection (#7).

**Figure 1:**
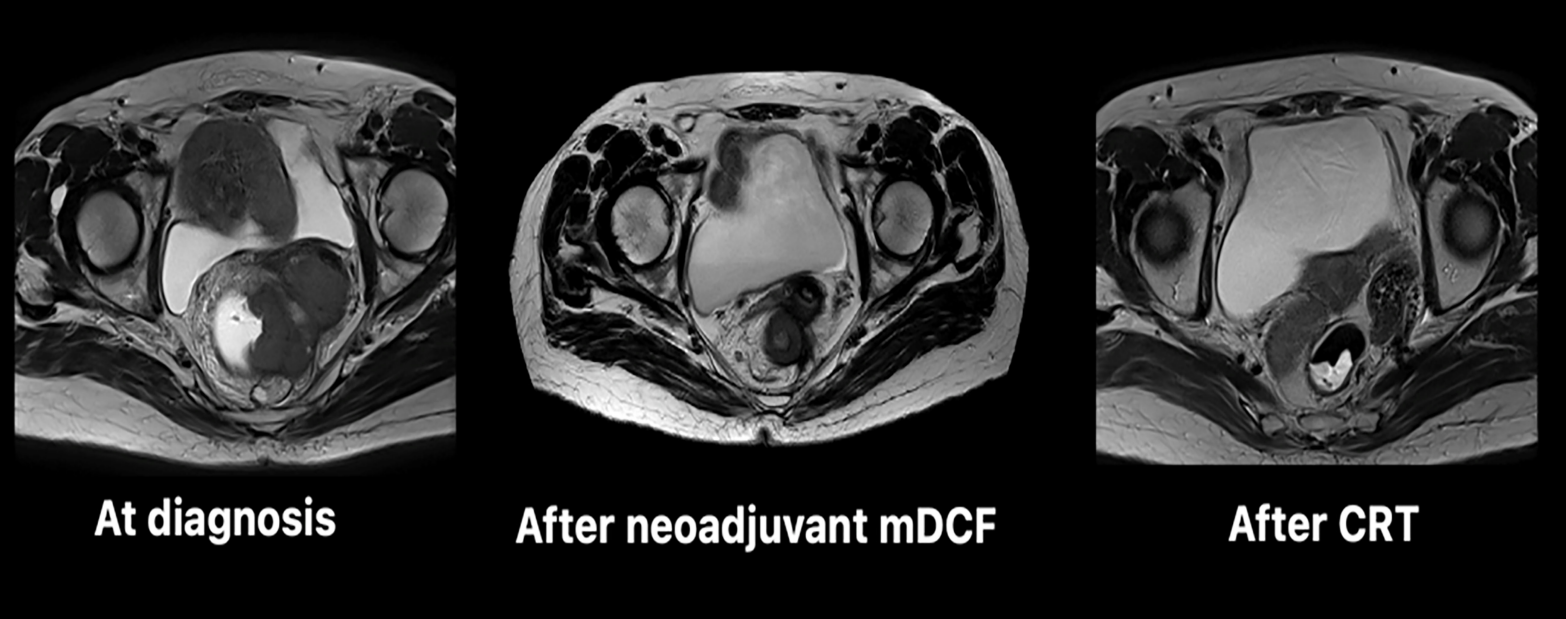
Evolution of the rectal squamous cell carcinoma followed by pelvic MRI during treatment (patient #1). CRT, chemoradiotherapy; mDCF, modified DCF chemotherapy.

Proctectomy with total mesorectal resection was performed for one patient assessed in complete response after mDCF neoadjuvant chemotherapy followed by CRT (#1, **Figure 1**). The pathological analysis confirmed a histological complete response. Of note, the post-operative complication was associated with a chronic pelvic fistula.

### Follow-up

After mDCF chemotherapy ± CRT strategy, one resistant tumor (#7), one locoregional relapse at 16 months (#3), and one distant relapse at two years (#8) were reported. Different oncologic management of these patients were applied. Patient #7 had a disease progression despite paclitaxel-based second-line chemotherapy. Patient #3 was treated with an immune checkpoint inhibitor targeting PD-L1 for two months and then by polychemotherapy combining methotrexate, cisplatin, and doxorubicin for six months. Patient #8 exhibited a lung metastasis treated by stereotaxic radiotherapy and is still in complete remission.

The median RFS was 20.7 months (95%CI=11.7-NA). After a median follow-up equal to 33.1 months (IQR, 15.5 – 52.8 months), 77.8% of patients were alive and without disease and the median OS was not reached at six years.

## Discussion

The prognosis of locally advanced rSCC is poor and one of the most important prognostic factors appears to be CRR after CRT (45). Previous studies reported a CRR in locally advanced diseases of around 57% versus 72% for all stages combined (**Table 3**). In our cohort, the addition of neoadjuvant chemotherapy with mDCF appears to improve the CRR to 75% in locally advanced diseases. This may also allow an increase in OS.

However, we observed more relapses than reported in previous studies (33.3% versus 13%) (26), but this difference can be explained by the almost exclusive presence of locally advanced and metastatic diseases in our cohort, whereas previous reports included patients with heterogeneous tumor characteristics (mostly locally advanced and localized diseases). The RFS and OS results are encouraging with a median RFS of 33.1 months and a median OS not reached in a population where the five-year OS rate in the literature is 30% (27).

Besides, increasing the CRR could also limit the indications for rectal surgery (abdominoperineal amputation or Low Anterior Resection). Thus, the achievement of a complete response might be a relevant clinical endpoint in rSCC. Organ preserving strategies are a major issue regarding rSCC patients’ quality of life but also regarding the potential adverse events occurring after abdominoperineal amputation or total mesorectal excision. Indeed, postoperative mortality ranged between 1 to 7%, while post-operative adverse events are reported in 13 to 46% of the patients (40, 44). Anastomotic fistulas occur in about 10% of cases and are more frequent when the tumor location is low (47, 48). Fecal incontinence is estimated to be around 30% (49) after sphincter preserving surgery.

In the literature, salvage surgery was necessary in about 30-50% of the cases (**Table 4**). In contrast, in our cohort, only one patient underwent surgery (#1) with a pathological complete response on the surgical specimen. The high CRR in our cohort seems to be even better than that with CRT alone. Therefore, our results showed that mDCF chemotherapy and CRT generate a high level of complete remission in advanced rSCC leading to organ preservation in most of the patients treated in this cohort.

The treatment of relapsing rSCC is another important unresolved issue. We first showed that mDCF is effective in patients with metastatic diseases or patients displaying a relapse after the previous CRT. Treatment of subsequent disease progression might rely on immune checkpoint inhibition. Lyford-Pike et al. (50) showed in HPV-related head and neck SCC a membrane expression of PD-L1 in epithelial cells, macrophages of tonsil crypts (initial site of HPV infection), and CD8^+^ T cells. Several second-line studies after chemotherapy (51, 52) have shown ORR around 10-20% including some complete remissions. Immunotherapy is therefore a possible option in patients who progress after chemotherapy. In our population, only one patient was exposed to immunotherapy with atezolizumab (anti-PD-L1) in the second line. No efficacy was observed, with evidence of progression at the first assessment.

One of the main strengths of this work is the homogeneity of the cohort and the management. Indeed, all patients had at least a tumor classified as T3 and only one patient had N0 disease at diagnosis. Moreover, all patients have been treated with the same management regardless of the center or the referring physician. However, there are several limitations. First and foremost the small number of patients and the retrospective nature of data.

Our results suggest that mDCF is effective in rSCC disease. High levels of tumor responses were observed in line with our previous results in aSCC. A second important observation provided here is the feasibility of rectal radiotherapy following mDCF chemotherapy. Indeed, no limiting toxicity was observed in rSCC patients exposed to CRT after mDCF. Altogether, advanced rSCC is a very rare gastrointestinal cancer. The high rates of organ preservation and RFS observed here suggest that rSCC might be treated such as aSCC with mDCF when organ preservation strategies are compromised in advanced disease setting.

## Data availability statement

The original contributions presented in the study are included in the article. Further inquiries can be directed to the corresponding author.

## Ethics statement

The studies involving human participants were reviewed and approved by National French Commission for bioinformatics data and patient liberty. Written informed consent for participation was not required for this study in accordance with the national legislation and the institutional requirements.

## Author contributions

CB, AV conceived the study. LH, CB, AV collected patient cohort. AV performed statistical analyses. LH, SK, JB, EK, MP, TN, ZL, CB, AV analyzed the data. LH, CB, AV wrote the manuscript. All authors contributed to the article and approved the submitted version.

## Acknowledgments

The authors would like to thank Guadalupe Inés Tizón for English writing assistance.

## Conflict of interest

The authors declare that the research was conducted in the absence of any commercial or financial relationships that could be construed as a potential conflict of interest.

## Publisher’s note

All claims expressed in this article are solely those of the authors and do not necessarily represent those of their affiliated organizations, or those of the publisher, the editors and the reviewers. Any product that may be evaluated in this article, or claim that may be made by its manufacturer, is not guaranteed or endorsed by the publisher.
